# Histopathological Spectrum and Demographic Features of Hydatid Disease: A Five-Year Study From a Tertiary Care Center in Southern Saudi Arabia

**DOI:** 10.7759/cureus.109169

**Published:** 2026-05-19

**Authors:** Sohaila Fatima, Tabish W Siddiqui, Raqshan W Siddiqui, Shiza W Siddiqui, Wajih A Siddiqui

**Affiliations:** 1 Pathology, King Khalid University, Abha, SAU; 2 Medicine, Emirates Health Services, Ras Al Khaimah, ARE; 3 Internal Medicine, King Khalid University Hospital, Abha, SAU; 4 Oncology, Aseer Central Hospital, Abha, SAU

**Keywords:** cystic echinococcosis, histopathology, hydatid disease, retrospective study, saudi arabia

## Abstract

Background

Hydatid disease (cystic echinococcosis) is a neglected zoonotic parasitic infection caused by the larval stage of *Echinococcus granulosus*. It remains endemic in livestock-raising regions, including the Middle East, where humans serve as accidental intermediate hosts. The liver and lungs are most commonly affected, although any organ may be involved. Histopathology remains the definitive diagnostic modality, particularly in atypical cases.

Methods

This retrospective observational study was conducted at a tertiary care center in southern Saudi Arabia over a five-year period (January 2018 to December 2022). All histopathologically confirmed cases of hydatid disease were included. Data on age, sex, and anatomical site were retrieved from pathology records. Diagnosis was based on characteristic microscopic features, including laminated acellular membranes, fibrous pericyst, and protoscolices or hooklets. Only descriptive statistics were used.

Results

A total of 18 cases were identified. The mean age was 39.4 years (range: 20-71 years), with a female predominance (n = 11, 61.1%). The liver was the most frequently involved organ (n = 11, 61.1%), followed by the lungs (n = 5, 27.7%). Rare involvement of the spleen and stomach was observed in one case each (n = 1, 5.6%). Histopathological examination consistently demonstrated the characteristic trilaminar structure of hydatid cysts, with protoscolices and hooklets identified in several cases.

Conclusion

Hydatid disease in this cohort predominantly involved the liver, followed by the lungs, with a female predominance and occasional atypical organ involvement. Histopathology remains essential for definitive diagnosis, particularly in unusual presentations. Awareness of its clinicopathological spectrum is important for early recognition in endemic regions.

## Introduction

Hydatid disease, also known as cystic echinococcosis, is a neglected zoonotic parasitic infection caused by the larval stage of *Echinococcus* species, most commonly *Echinococcus granulosus *[1]. It remains a significant global public health concern in endemic regions where close interaction between humans, livestock, and domestic dogs facilitates sustained transmission. The parasite is maintained in a biological cycle involving canines as definitive hosts and herbivorous animals, particularly sheep and cattle, as intermediate hosts [1]. Humans become accidental intermediate hosts following ingestion of parasite eggs through contaminated food, water, soil, or direct contact with infected dogs.

After ingestion, the eggs release oncospheres that penetrate the intestinal mucosa and enter the portal circulation [2]. The liver acts as the first major filter, trapping most larvae, while some escape into systemic circulation and lodge in the lungs or other organs. Over time, these larvae develop into slow-growing hydatid cysts, which may remain clinically silent for years or even decades [2].

Epidemiologically, hydatid disease is widely distributed in regions where livestock farming is common, including the Middle East, Mediterranean basin, Central Asia, South America, and parts of Africa [3]. In Saudi Arabia, the disease continues to be encountered due to traditional animal-rearing practices, rural exposure, and insufficient control of stray dog populations [4]. The disease predominantly affects young and middle-aged adults, who are more likely to be exposed through occupational or household activities [1]. Several studies have also reported a mild female predominance, potentially related to differences in domestic exposure patterns [1,2].

Clinically, hydatid disease is characterized by a long latent period, and most patients remain asymptomatic until cysts reach a significant size or cause complications [1]. Clinical manifestations depend on the organ involved, cyst size, and the presence of complications, such as rupture, secondary infection, or mass effect on adjacent structures [2]. The liver is the most commonly affected organ, followed by the lungs; however, hydatid cysts may develop in virtually any organ system, including the spleen, kidneys, brain, bones, and gastrointestinal tract [2]. Patients may present with non-specific symptoms, such as abdominal pain, hepatomegaly, cough, chest pain, dyspnea, or hemoptysis, while many cases are incidentally detected during imaging for unrelated conditions. Complicated hydatid disease may lead to serious outcomes. Cyst rupture can result in secondary dissemination within the peritoneal or pleural cavities and may also trigger life-threatening hypersensitivity reactions, including anaphylactic shock [1,2]. These potential complications make early recognition and accurate diagnosis essential for preventing morbidity.

From a diagnostic perspective, hydatid disease remains challenging due to its ability to mimic benign cysts, abscesses, or neoplastic lesions [5]. Imaging modalities, such as ultrasound, computed tomography (CT), and magnetic resonance imaging (MRI), are central to detection and staging. Serological tests may provide supportive evidence; however, their sensitivity varies depending on cyst location and viability [1]. Consequently, histopathological examination remains the definitive diagnostic modality, particularly in atypical or surgically resected cases. Histologically, hydatid cysts demonstrate a distinctive multilayered structure composed of an outer host-derived fibrous pericyst, a middle laminated acellular membrane, and an inner germinal layer responsible for producing brood capsules and protoscolices [6]. The presence of hooklets, scolices, or daughter cysts further confirms the diagnosis.

Despite its continued clinical relevance, there is a paucity of detailed histopathological studies from southern Saudi Arabia describing the full spectrum of organ involvement, including atypical presentations. Understanding local disease patterns is essential for improving diagnostic awareness and guiding clinical suspicion. Therefore, this retrospective study aims to evaluate the histopathological and demographic features of hydatid cyst cases diagnosed over a five-year period at a tertiary care center in southern Saudi Arabia, with emphasis on age, sex distribution, organ involvement, and microscopic characteristics.

## Materials and methods

This retrospective descriptive study was conducted at a tertiary care center in southern Saudi Arabia. The study covered a five-year period from January 2018 to December 2022.

All cases diagnosed histopathologically as hydatid cyst disease during the study period were included. Cases were retrieved through electronic pathology database search and archival histopathology records using the diagnostic terms "hydatid cyst" and "echinococcosis." Only histopathologically confirmed cases with adequate tissue sections and complete demographic information were included in the analysis. Cases with insufficient histological material or incomplete records were excluded. Available radiological reports were reviewed in selected atypical cases for clinicoradiological correlation.

Tissue samples consisted of formalin-fixed, paraffin-embedded sections processed routinely in the histopathology laboratory. Hematoxylin and eosin (H&E) staining was used in all cases. Tissue sections of approximately 3-5 µm thickness were prepared from formalin-fixed paraffin-embedded blocks using standard microtomy techniques. Routine H&E staining protocols were performed according to laboratory practice. Histopathological examination and photomicrograph acquisition were performed using standard light microscopy under multiple magnifications. Diagnosis was confirmed based on the presence of laminated acellular membranes, fibrous host reaction (pericyst), and, when present, protoscolices, hooklets, or daughter cyst structures.

Demographic data, including age, sex, and anatomical site of involvement, were extracted from laboratory records. The anatomical distribution of hydatid disease was categorized into organ-based groups for analysis.

This study utilized anonymized archival histopathology specimens and laboratory records with no direct patient contact or identifiable personal information. Therefore, formal ethical approval was not applicable. All data were handled confidentially in accordance with institutional standards.

Data analysis was performed using descriptive statistics. Continuous variables, such as age, were expressed as mean, median, and range, while categorical variables, such as sex and site distribution, were presented as frequencies and percentages. Statistical analysis was performed using Statistical Product and Service Solutions (SPSS, version 25.0; IBM SPSS Statistics for Windows, Armonk, NY).

## Results

A total of 18 histopathologically confirmed cases of hydatid disease were identified during the five-year study period. The patients ranged in age from 20 to 71 years, with a mean age of 39.4 years, reflecting a predominance of young to middle-aged adults. The age distribution showed a peak incidence in the third and fourth decades of life, with a gradual decline in older age groups.

There was a female predominance, with 11 females (61.1%) and seven males (38.9%), resulting in a female-to-male ratio of 1.6:1. The demographic characteristics are summarized in Table 1. 

**Table 1:** Demographic characteristics of patients with hydatid disease

Characteristic	Value (n)	Percentage (where applicable) (%)
Male	7	38.9%
Female	11	61.1%
Total	18	100%
Mean age	39.4 years	
Age range	20-71 years	

**Figure 1:**
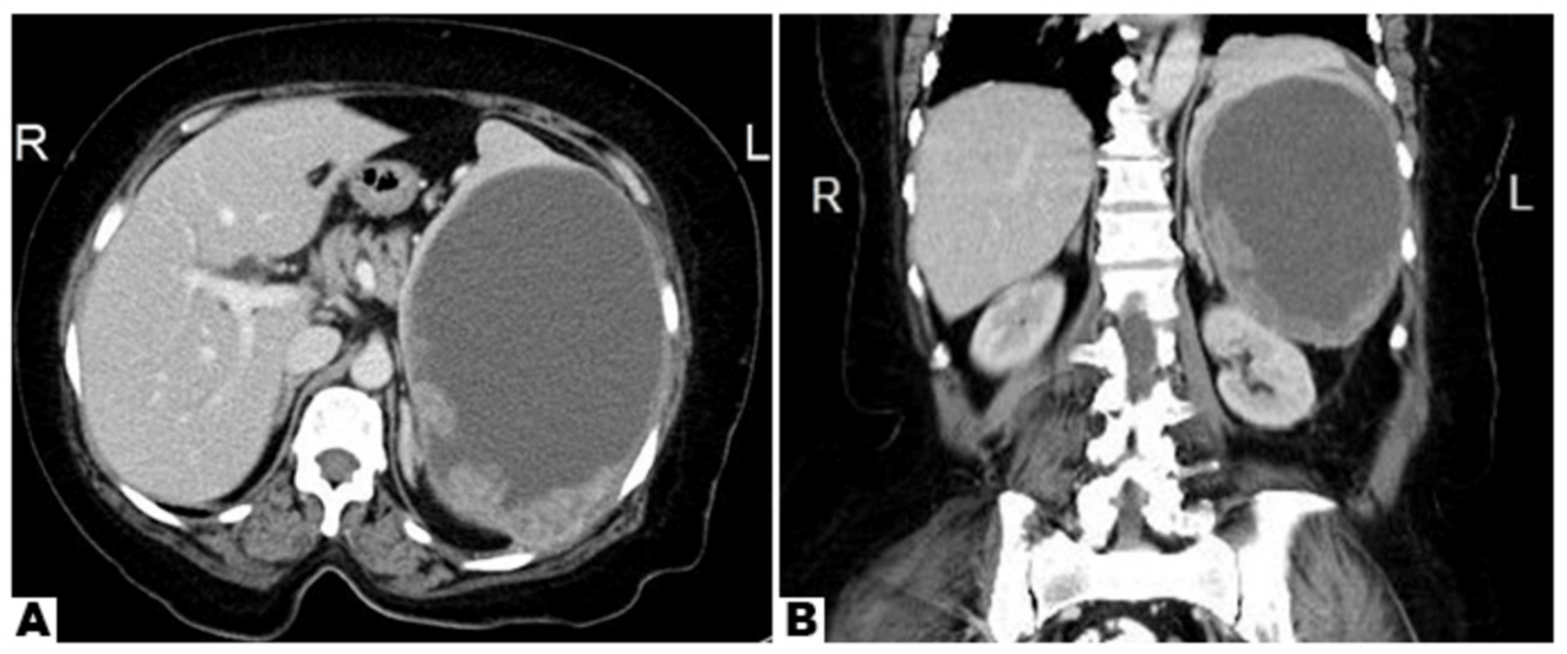
Contrast-enhanced CT scan of the abdomen (A) Axial and (B) coronal view showing a large unilocular thick-walled splenic cyst, consistent with splenic hydatid disease.

The liver was the most frequently involved organ, accounting for 11 cases (61.1%). The lungs were the second most common site of involvement, observed in five cases (27.7%). Rare involvement of the spleen and stomach was noted in one case each (5.6%). Published literature indicates that atypical organ involvement in hydatid disease is relatively uncommon compared with hepatic and pulmonary disease. Splenic involvement has been reported in approximately 0.5%-4% of cases, while gastric involvement is exceedingly rare and is usually described in isolated case reports or small series [7]. Therefore, the frequency observed in our cohort falls within the reported range and likely reflects the relatively small sample size rather than a true increase in prevalence. Nevertheless, these findings reinforce the broad anatomical spectrum of hydatid disease and highlight the importance of considering hydatid cysts in the differential diagnosis of unusual cystic lesions. In the splenic case, CT imaging demonstrated a large unilocular thick-walled splenic cyst measuring 15.9 × 10.3 × 14.5 cm (Figure 1).

The anatomical distribution of cases is shown in Table 2.

**Table 2:** Anatomical distribution of hydatid cyst cases

Site	Frequency (n)	Percentage (%)
Liver	11	61.1%
Lung	5	27.7%
Spleen	1	5.6%
Stomach	1	5.6%

Age-wise stratification revealed that hepatic hydatid disease was most common among patients aged 21-50 years, while pulmonary cases were observed across a wider age distribution. The atypical cases involving the spleen and stomach occurred in middle-aged individuals, highlighting the potential for extrahepatic and extrapulmonary involvement in routine clinical practice.

Histopathological examination consistently demonstrated the classical features of hydatid disease in all cases. All specimens revealed a laminated acellular membrane surrounded by a fibrous pericyst formed by host inflammatory response. The germinal layer was identified in multiple cases, with evidence of brood capsule formation and protoscolices in viable cysts. Hooklets and fragmented parasitic structures were also observed, confirming the diagnosis. Degenerative changes, such as necrosis and collapse of cyst architecture, were seen in a subset of long-standing lesions.

Organ-specific histological differences were noted. Hepatic cysts showed more prominent fibrous host reaction compared to pulmonary lesions, which exhibited relatively thinner fibrous walls. Atypical site involvement retained the same basic histological architecture despite degenerative changes.

**Figure 2:**
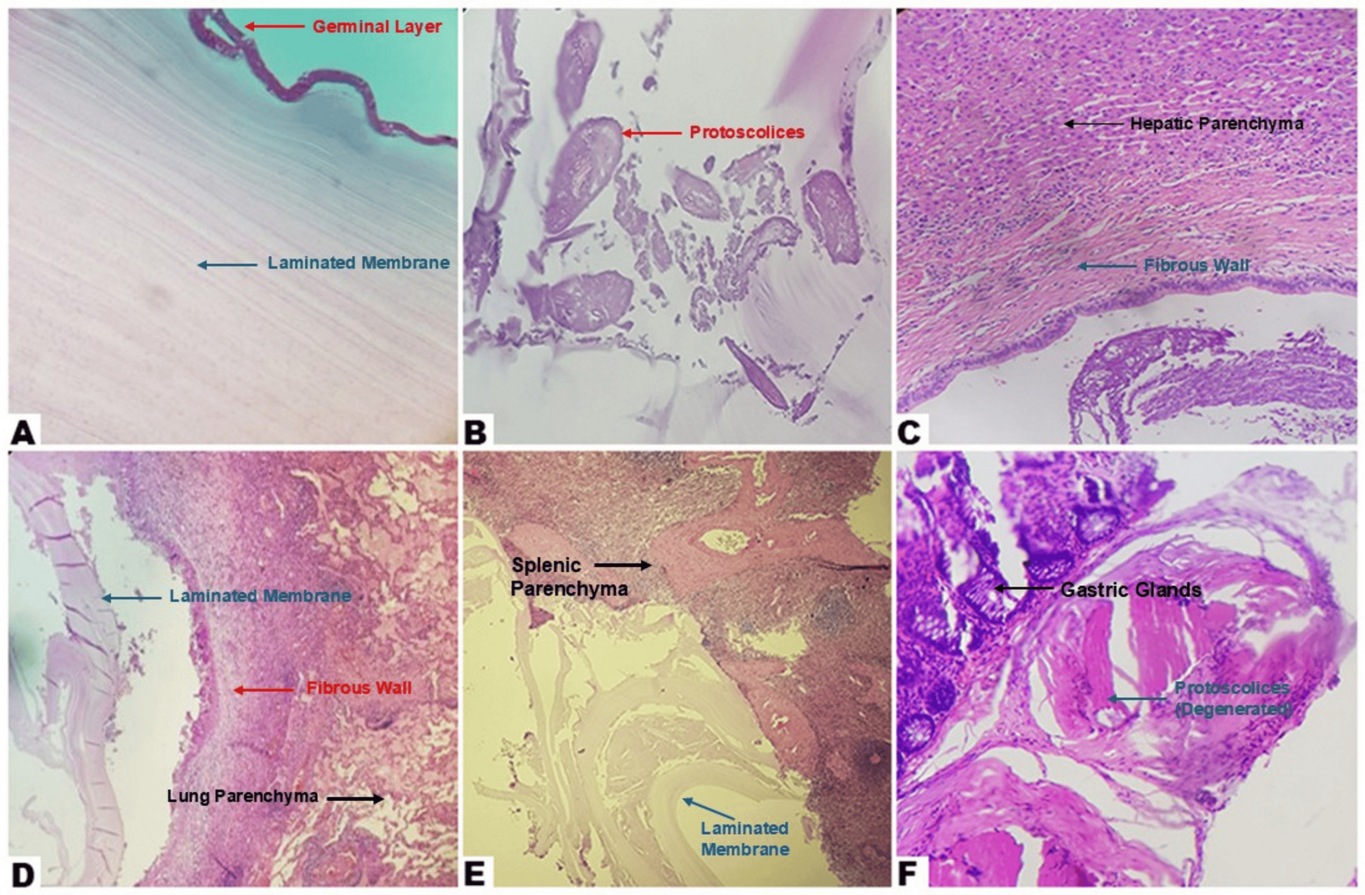
Histopathological features of hydatid disease in different organs (hematoxylin and eosin stain) (A) Laminated acellular membrane (blue arrow) and germinal layer (red arrow) (20x magnification). (B) Necrotic protoscolices (red arrow) within the cyst cavity (40x magnification). (C) Fibrous (blue arrow) pericyst in hepatic (black arrow) hydatid disease (10x magnification). (D) Pulmonary (black arrow) hydatid cyst showing laminated membrane (blue arrow) and fibrous wall (red arrow) (10x magnification). (E) Splenic (black arrow) hydatid cyst with characteristic cyst wall architecture showing laminated membrane (blue arrow) (10x magnification). (F) Gastric (black arrow) hydatid cyst with degenerated protoscolices (blue arrow) (20x magnification).

Representative histopathological features are illustrated in Figure 2.

## Discussion

Hydatid disease remains an important parasitic infection in endemic regions, particularly where livestock farming and close human-animal interaction are common [8]. The present study provides a histopathological overview of hydatid disease over a five-year period in southern Saudi Arabia, highlighting its demographic profile, organ distribution, microscopic characteristics, and the continued relevance of tissue diagnosis. The disease is acquired by ingestion of eggs shed in dog feces, and human infection is accidental; once the oncospheres are swallowed, they pass through the portal circulation and most commonly lodge in the liver, with the lungs as the next most frequent site [9].

The female predominance in our study is consistent with several reports from endemic settings, although sex distribution varies across geographic populations and exposure settings [10-12]. This variation may reflect local exposure patterns described in previous studies, including household responsibilities, food preparation, animal contact, and differences in rural lifestyle rather than any fixed biological susceptibility; however, our study did not directly assess occupational or behavioral risk factors. The mean age of 39.4 years and concentration of cases in young to middle-aged adults are also in keeping with the slow-growing nature of hydatid cysts, which often remain asymptomatic for years before diagnosis [3].

The predominance of hepatic involvement in our cohort (61.1%) is consistent with established pathophysiology and published literature [13]. The liver acts as the first filter in the portal circulation, trapping most larvae, while pulmonary involvement reflects passage through the hepatic filter and subsequent lodging in the lungs [14]. Large reviews note that liver involvement typically accounts for about 50%-70% of cases, with the lungs representing the second most common site [13]. Our findings are therefore in line with the expected organ distribution reported in endemic populations.

Although less common, the presence of splenic and gastric hydatid cysts in our series is clinically important because atypical sites can mimic benign cysts, abscesses, or neoplastic lesions [1]. Such presentations may delay diagnosis if hydatid disease is not considered in the differential, especially in nonhepatic or extrapulmonary locations. The recognition of these uncommon sites is particularly important for pathologists and surgeons working in endemic regions, where hydatid disease may involve almost any organ.

Histopathological examination remains the cornerstone of definitive diagnosis in resected or biopsied specimens [6]. The characteristic laminated acellular membrane, germinal layer, and host-derived fibrous reaction are diagnostic, and the presence of protoscolices or hooklets provides further confirmation [15]. In our study, histopathology confirmed all cases, reinforcing its value even in the era of advanced imaging. This is especially relevant in unusual sites or complicated lesions, where radiologic appearances may not be specific enough to establish a final diagnosis.

Management of hydatid disease is individualized and depends on cyst location, stage, size, complications, and available expertise [16]. Treatment options include albendazole-based medical therapy, percutaneous procedures, such as puncture, aspiration, injection, re-aspiration (PAIR), and surgery for complicated, large, superficial, ruptured, or diagnostically uncertain cysts [16]. Imaging is central to treatment planning because ultrasound and cross-sectional imaging help stage the cyst and determine whether medical, percutaneous, or surgical treatment is most appropriate [17]. In practice, histopathology often complements management by confirming the diagnosis after resection or biopsy, especially in cases with atypical presentation or intraoperative uncertainty.

The main limitations of this study are its retrospective design, single-center setting, and small sample size, which limit generalizability and statistical inference. We also lacked standardized radiological staging data, comprehensive serological findings, treatment details, and long-term follow-up outcomes, limiting full clinicoradiological and prognostic correlation. In addition, the retrospective nature of the study depended on archival records, which may introduce selection and documentation bias. Despite these limitations, the study adds valuable regional data from southern Saudi Arabia and supports continued awareness of both typical and atypical manifestations of hydatid disease in endemic settings.

## Conclusions

Hydatid disease continues to be a significant parasitic infection in endemic regions, demonstrating a consistent predilection for hepatic involvement, followed by pulmonary disease. In this five-year histopathological study, most cases occurred in young to middle-aged adults, with a female predominance and occasional involvement of uncommon sites, such as the spleen and stomach. The presence of atypical organ involvement highlights the need to consider hydatid disease in the differential diagnosis of cystic lesions in virtually any anatomical location, particularly in endemic settings. Recognition of these patterns is essential to avoid misdiagnosis and to ensure timely clinical intervention.

Histopathological examination remains the definitive diagnostic modality, especially in cases where imaging is inconclusive or when lesions arise in unusual sites. The characteristic microscopic features provide reliable confirmation and remain indispensable in routine diagnostic practice. Overall, this study reinforces the typical histopathological spectrum and demographic distribution of hydatid disease in southern Saudi Arabia and underscores the importance of maintaining a high index of suspicion for both common and atypical presentations.
